# Whole‐exome sequencing identified compound heterozygous variants in *ROR2* gene in a fetus with Robinow syndrome

**DOI:** 10.1002/jcla.23074

**Published:** 2019-10-16

**Authors:** Kai Yang, Jianjiang Zhu, Ya Tan, Xiaofei Sun, Huawei Zhao, Guodong Tang, Dongliang Zhang, Hong Qi

**Affiliations:** ^1^ Department of Obstetrics and Gynecology Peking University International Hospital Beijing China; ^2^ Department of Prenatal Diagnosis Center Haidian Maternal and Child Health Care Hospital Beijing China; ^3^ Department of Orthodontics School of Stomatology Capital Medical University Beijing China

**Keywords:** immunohistochemistry, Robinow Syndrome, *ROR2* gene, western blotting, whole‐exome sequencing

## Abstract

**Background:**

Autosomal recessive Robinow syndrome (ARRS) is a rare genetic disorder, which affects the development of multiple systems, particularly the bones.

**Objectives:**

The aim of this study was to investigate the genetic cause of a ARRS fetus and to evaluate the reliability of whole‐exome sequencing (WES) in prenatal diagnosis on cases with indistinguishable multiple malformation.

**Methods:**

Clinical and ultrasonic evaluations were conducted on the fetus, and multiplatform genetic techniques were used to identify the variation responsible for RS. The pathogenicity of the novel variation was evaluated by in silico methods. Western blotting (WB) and immunohistochemistry (IHC) were performed on fetal tissues after the fetus' stillbirth and postabortal autopsy.

**Results:**

A compound heterozygous variation consisting c.613C > T and c.904C > T in *ROR2* gene was identified. In silico prediction suggested that c.904C > T was a deleterious variant. IHC result demonstrated that *ror2* expression level of the proband in osteochondral tissue significantly increased comparing with that of the control sample.

**Conclusions:**

For the first time in Chinese population, we characterized a novel variation in *ROR2* gene causing ARRS. This study extended the mutation spectrum of ARRS and provided a promising strategy for prenatal diagnosis of cases with ambiguous multiple deformities.

## 1 INTRODUCTION

Robinow Syndrome (RS), firstly described by Robinow et al,^1^ is a rare genetic disorder characterized by dysmorphic craniofacial features resembling a "fetal face," mesomelic limb shortening, kyphoscoliosis, hemivertebrae, hypoplastic external genitalia, and renal anomalies.^2^ Both autosomal dominant and autosomal recessive patterns of inheritance have been reported. Among which, *ROR2*‐related Robinow Syndrome (ARRS; MIM #268310) is the autosomal recessive form caused by pathogenic variations in the *ROR2* (MIM *602337) gene located at 9q22. Symptoms of ARRS are more severe than those of autosomal dominant RSs (ADRS; MIM #180700, #616331, and #616894) caused by *WNT5A* (MIM *164975), *DVL1* (MIM *601365), or *DVL3* (MIM *601368).^3^, ^4^, ^5^ So far, less than 200 cases of ARRS have been reported, mainly in consanguineous families, for example, those of Turkish, Omani,^6^ and Egyptian^7^ origin.

The human *ROR2* gene comprises nine exons and eight introns, including 226kp bases. Thus far, 26 mutations associated with ARRS have been reported in the *ROR2* gene (http://101.200.211.232/skeletongenetics/), most of which are stopgain or deletion variations in the 5‐9 exons.

Herein, we described the clinical, anatomical, and molecular findings of a fetus with typical ARRS manifestation from a non‐consanguineous couple with normal phenotype. Two heterozygous variants were identified including a novel one in the *ROR2* gene. Moreover, the results of *ror2* protein expression detection were consistent with this finding, which confirmed the pathogenicity of this variation.

## 2 MATERIALS AND METHODS

### 2.1 Subjects

This study was approved by the Ethics Committee of Haidian Maternal and Child Healthcare Hospital (No. 2018‐10), and informed consent was signed by the recruited couple.

**Figure 1:**
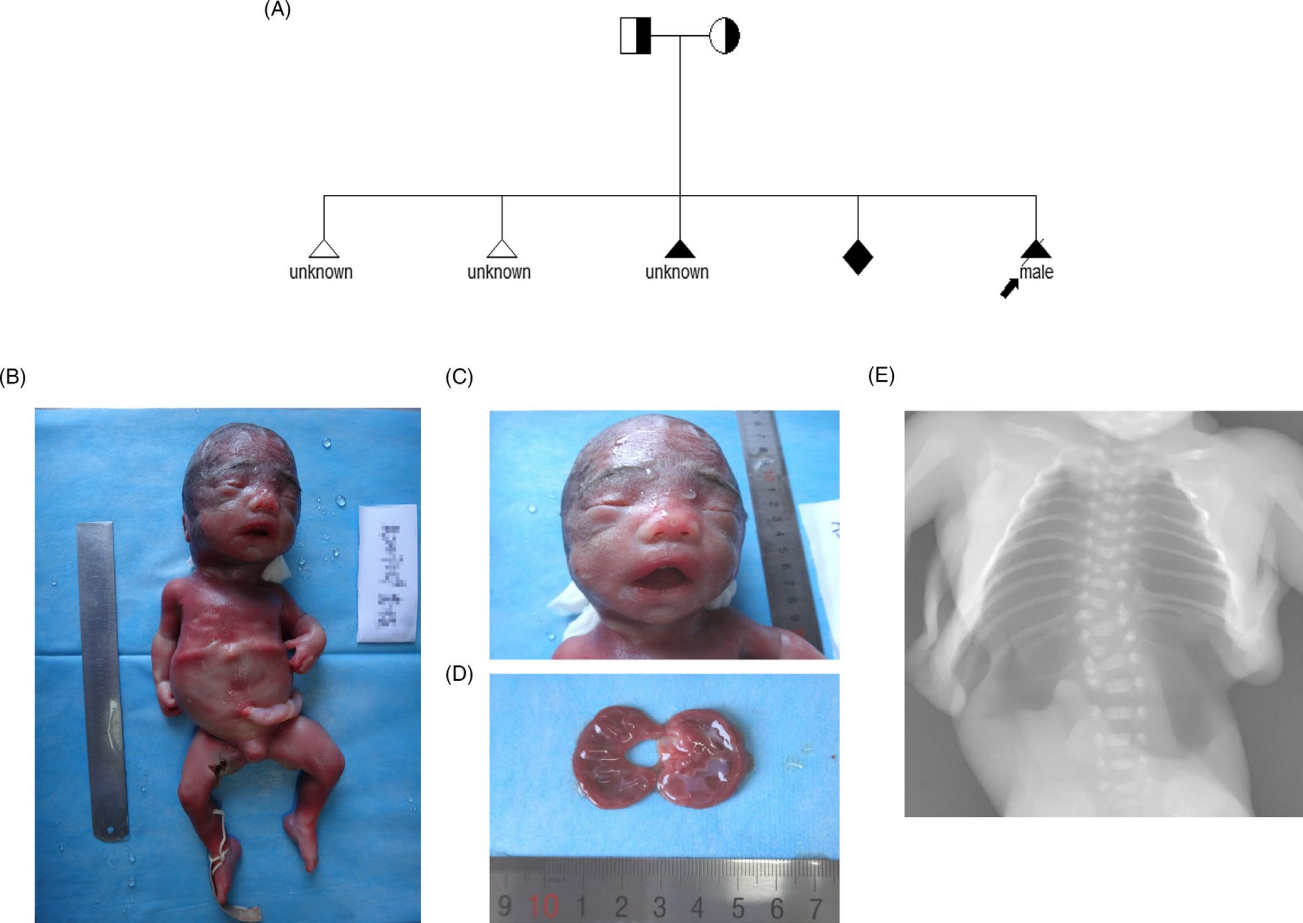
Clinical data of the case. A, The pedigree of this case; B, the front image of the proband; C, the special facial features; D, the renal cystic degeneration of the proband; and E, the vertebrae and rib abnormalities in X‐ray image

A 39‐year‐old pregnant women gravida 5 para 0 referred to the center of prenatal diagnosis in Haidian Maternal and Child Healthcare Hospital. She and her husband had been through 4 times of adverse pregnancies including twice spontaneous abortions at first trimester and twice pregnancies with multiple malformation terminated at 23 and 25 gestational weeks, respectively (Pedigree shown in Figure 1). Conventionally, amniocentesis was conducted at 19 weeks of gestation. Afterwards, ultrasonography revealed multiple malformations in the fetus at 22 weeks of gestation. Fetal stillbirth occurred at 24^+2^ weeks, thus, induced labor operation was conducted.

### 2.2 Genetic analysis

Conventional karyotying by G‐banding and fluorescence in situ hybridization (FISH) were performed on amniotic fluid cells (AFCs) according to standard operation procedures to detect overall chromosomal anomalies. Chromosomal microarray analysis (CMA) with CytoScan 750K SNP Array(Affymetrix Inc) was conducted according to the manufacturer's manual workflow on DNA extracted from AFCs, so that to investigate genomic copy number variants with clinical significance. Data were collected and analyzed by GeneChip Scanner 3000 with AGCC software.

After induced abortion, more DNA was extracted from umbilical cord sample and then used for WES detection. DNA fragments hybridization (by IDT’s xGen Exome Research Panel, Integrated DNA Technologies), library quality testing, and WES sequencing (using Novaseq6000 platform, Illumina) were performed as described in our previous study.^8^ Sanger sequencing was used to verify the variation of suspected pathogenicity. The conservation of specific amino acid in various species was obtained from NCBI blast (https://blast.ncbi.nlm.nih.gov/Blast.cgi). The pathogenicity index of specific variant was analyzed using Sorting Intolerant From Tolerant (SIFT) (http://sift.bii.a-star.edu.sg/) and Polymorphism Phenotyping V2 (http://genetics.bwh.harvard.edu/pph2/).

### 2.3 Western blotting and IHC

The body of the fetus was sent for pathological examination. Then, the fetal skin tissue sample from the proband was used for WB testing, along with a *ROR2* wild type sample at similar gestational age as normal control. Meanwhile, the paraffin sections of fetal auricular finger osteochondrocytes were subjected to IHC detection, along with a costal cartilage tissue sample from an aborted fetus (*ROR2* wild type) at similar gestational age as normal control. Both of these experiments were performed with monoclonal anti‐*ror2* antibody (#ab190145, Abcam; with beta Actin antibody #119716 as inner control). All experimental procedures were carried out according to the manufacturer's protocols (https://www.abcam.com/ror2-antibody-nt-2535-2835-ab190145.html). Image processing was performed with Image J software. Statistical analysis was performed with Prism 6 software (GraphPad). Two‐tailed Student's *t* test was used to determine the difference between two groups.^9^

## 3 RESULTS

### 3.1 Clinical data

The result of ultrasonographic diagnosis at 22 weeks indicated that the proband fetus had multiple structural abnormalities, including micrognathia, rachioscoliosis, limbs shortening, and genital abnormality. Pathological examination result suggested that the anomalies of the fetus were: macrocephaly, frontal bossing, splay eyebrows, ocular hypertelorism, depressed nasal bridge, anteverted nares, inverted V‐shaped mouth, micrognathia, low‐set ears; mesomelia; external genital dysplasia; scoliosis, thoracic 9, 12 hemivertebra, thoracic 2, 3, 4, 5, 7, and 11 vertebra dysplasia, thoracic 10 vertebra loss, left rib 8, 9, and 10 partial fusion deformity, right rib 9 and 10 fusion deformity; and bilateral polycystic kidneys (Figure 1).

### 3.2 Results of genetic analysis

**Figure 2:**
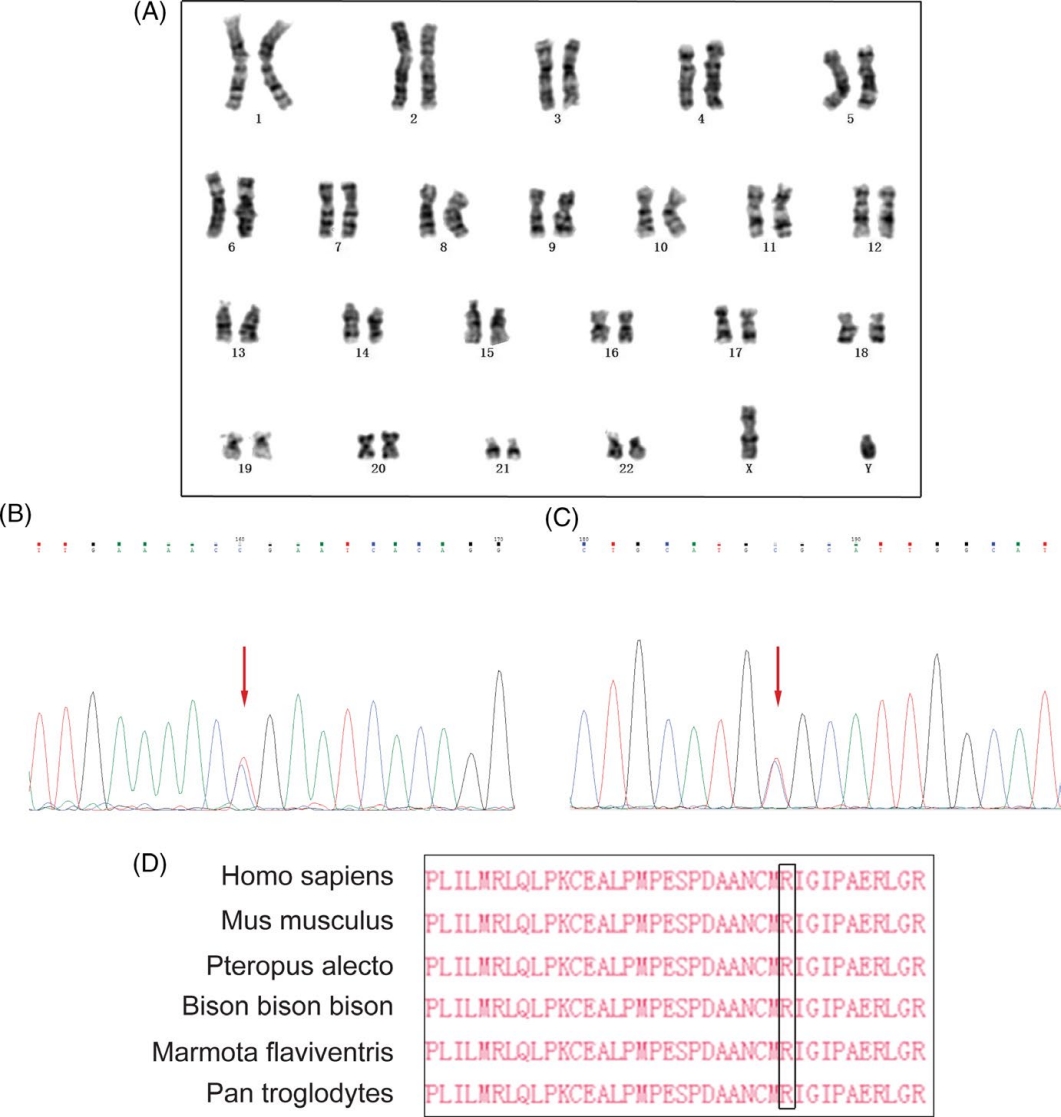
Results of genetic analysis. A, The chromosome karyotype of the proband; B, Sanger sequencing showing a nucleotide substitution (ROR2: c.613C > T); C, a nucleotide substitution (ROR2: c.904C > T); and D, the conversation status of ROR2: Arg302 acid among multiple mammal species

Results of karyotyping and CMA were both normal (Figure 2A).

Two compound heterozygous variants in *ROR2* comprising c.613C > T(p.Arg205*) and c.904C > T(p.Arg302Cys) were identified by WES and confirmed by Sanger sequencing (Figure 2B and C). In addition, Sanger sequencing also revealed that the father was a carrier of heterozygous c.613C > T, while the mother was a carrier of heterozygous c.904C > T. As a novelly detected variant with potential pathogenesis, c.904C > T has a very low frequency in variation databases, namely 0 in 1000 Genome (https://www.internationalgenome.org/) and 1.772e‐05 in ExAC (http://exac.broadinstitute.org/variant/9-94495436-C-T). The *ROR2*: Arg302 amino acid among mammal species is highly conserved (Figure 2D). Besides, prediction results from SIFT and PolyPhen V2 both suggested that c.904C > T was a deleterious missense variation.

### 3.3 Results of WB and IHC

**Figure 3:**
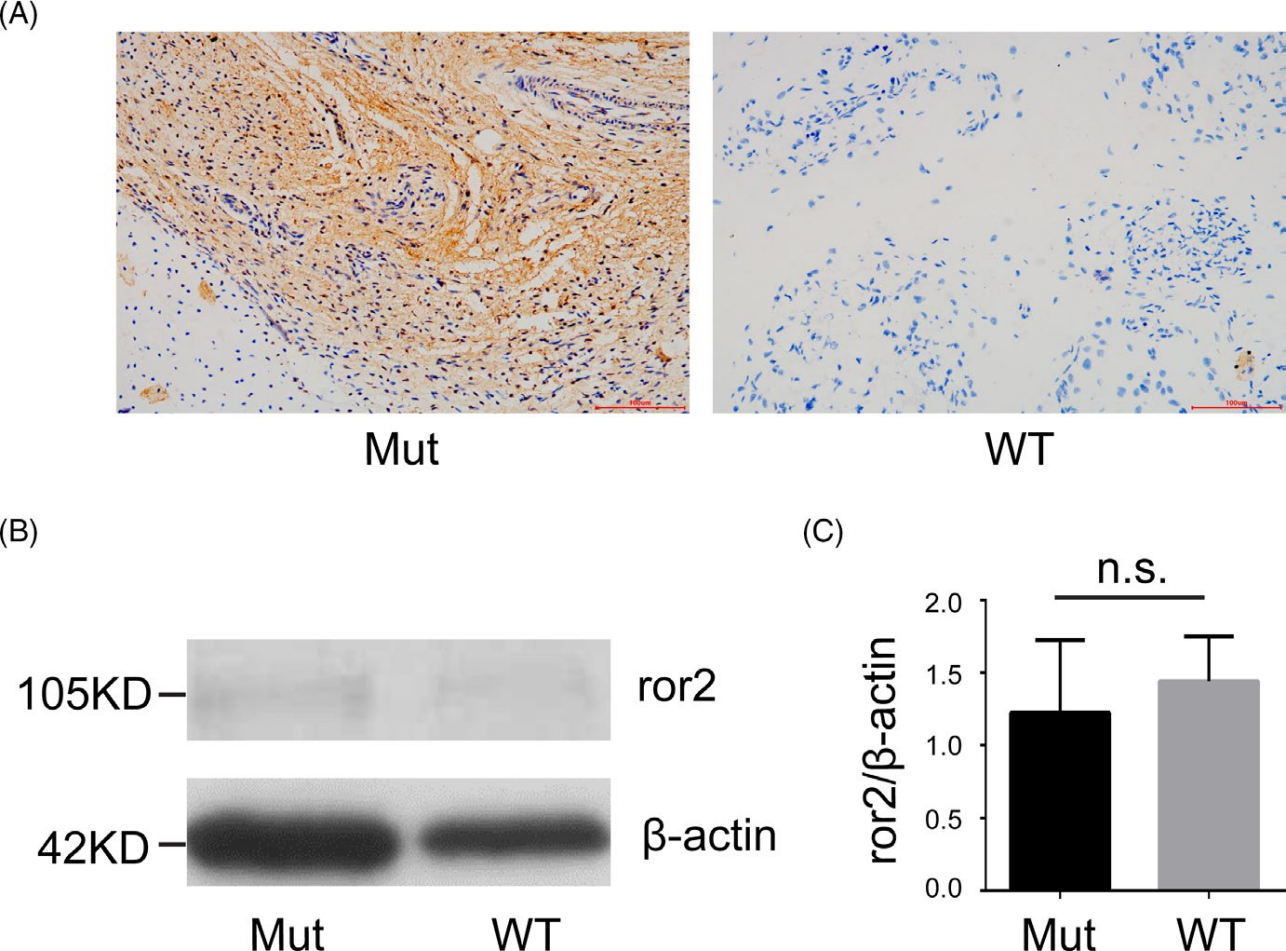
IHC and WB results. A, IHC results of the *ror2* expression in osteochondral tissues of the mutant(left) and wild type(right); B, WB results of the *ror2* expression in skin tissues of the mutant(left) and wild type(right); C, quantitative analysis of *ror2*/beta‐actin between mutant(left) and wild type(right) showed no significant difference

Immunohistochemistry result indicated that the expression level of *ror2* protein was significantly increased in the osteochondral tissues of the proband (Figure 3A). However, as shown in Figure 3B and C, Western blotting result demonstrated that the *ror2* expression level difference between the proband and wild type in skin tissue was not significant (*P* = .656).

## 4 DISCUSSION

As a type of severe and rare disorder involving skeletal dysplasia, Robinow syndrome has strong clinical and genetic heterogeneity and is difficult to distinguish from other semblable conditions. In this study, the manifestations of the proband were consistent with previous reports,^1^, ^10^, ^11^, ^12^, ^13^ so it can be clinically diagnosed as Robinow syndrome. However, the fetus ended up stillborn, which is not very common in ARRS cases, and the reason is still unclear. It may be due to the visceral extrusion caused by the abnormal thoracic development.

All genes that have been identified to cause this disoder, whether in an autosomal dominant or recessive pattern, are in the *Wnt* signaling pathway and interact strongly with each other.^4^, ^5^, ^14^ *ROR2*, the causative gene of ARRS, is a member of the receptor tyrosine kinase‐like gene family and encodes an orphan membrane‐bound tyrosine kinase which acts as a co‐receptor of *WNT5A* in the process of osteoclastogenesis.^15^ The *ROR2* transmembrane protein comprises several distinct motifs including immunoglobulin‐like (Ig), cysteine‐rich (CRD), and kringle domains in the extracellular region; a transmembrane section; and an intracellular region with tyrosine kinase (TK), serine/threonine‐rich, and proline‐rich structures.^16^ In an initial study, *ROR2* gene was reported to cause brachydactyly type B (BDB1, MIM #113000)^17^; Then, Ali et al first described that homozygous missense mutations in *ROR2* proteins, both intracellular and extracellular, and homozygous nonsense mutations that caused truncation at downstream of TK domain can lead to ARRS.^12^ The compound heterozygous variation detected in our study contains two variants. Among which, c.613C > T has been reported by van Bokhoven et al,^13^ and is a definite pathogenic variant; on the other hand, c.904C > T located in the frizzled domain (cysterine‐rich domain) was a novel missense variation, which has not been reported. According to the ACMG variant interpretation criteria,^18^ we determined it as a likely pathogenic variation with evidence level of PM2 + PM3+PP2 + PP3+PP4.

Ali et al^19^ and Mehawej et al^20^ demonstrated that mutations in the frizzled domain led to loss of function on account of retention of the mutated *ror2* protein inside the endoplasmic reticulum (ER) due to misfolding. The protein is then degraded using ER‐associated protein degradation and does not reach the plasma membrane. Thus, it was speculated that the effect of c.904C > T on *ror2* protein should conform to this mechanism. It was also reported that *ror2* expressed at high levels during early embryonic development, then the expression levels dropped strongly around day 16 and there were only very low levels in adult tissues (https://www.abcam.com/ror2-antibody-nt-2535-2835-ab190145.html). In this case, the expression levels of *ror2* protein in the fetus' osteoid cartilage were abnormally elevated at 24 weeks of gestation, most likely due to missense mutation activating the expression of one allele in the presence of an early terminated translation of the other allele that disrupted gene function. Yet, the mechanism of the abnormal high expression of the missense allele and its effect on the downstream molecules in its pathway still need further study. Moreover, there was no difference between the expression of *ror2* in skin samples of the proband and the control, indicating that the abnormal high expression may have tissue specificity. In addition, many studies pointed out that *ROR2* gene was highly expressed in a variety of malignant tumors^21^, ^22^, ^23^ and was listed as a potential target for treatment.^24^ Therefore, our study may provide an idea for the research on the treatment and mechanism of these malignant tumors.

Since the couple in this case were both carriers of pathogenic *ROR2* variants, their future pregnancy still has a 25% risk of being affected. So, their subsequent pregnancies should be followed by genetic counselling, and family plan options such as prenatal diagnosis, preimplantation genetic testing, or gamete donation should be considered.

At the present stage, the time and economic cost of WES have been profoundly reduced, the interpretation ability of its results has been gradually improved, and the corresponding disease databases have been continuously improved, all of which provide a prerequisite for its application in prenatal diagnosis in the near future.

In conclusion, we identified a novel heterozygous compound variation in *ROR2* gene leading to ARRS, which expanded the mutation spectrum of the disease. The high efficiency and sensitivity of WES are very helpful in identifying genetic causes of fetal congenital malformations with complex and ambiguous phenotypes.

## References

[1] Robinow M , Silverman FN . A newly recognized dwarfing syndrome. Am J Dis Child. 1969;117(6):645‐651. 5771504 10.1001/archpedi.1969.02100030647005

[2] Patton MA . Robinow syndrome. J Med Genet. 2002;39:305‐310. 12011143 10.1136/jmg.39.5.305 PMC1735132

[3] Person AD , Beiraghi S , Sieben CM , et al. WNT5A mutations in patients with autosomal dominant Robinow syndrome. Dev Dyn. 2010;239(1):327‐337. 19918918 10.1002/dvdy.22156 PMC4059519

[4] White J , Mazzeu JF , Hoischen A , et al. DVL1 frameshift mutations clustering in the penultimate exon cause autosomal‐dominant Robinow syndrome. Am J Hum Genet. 2015;96(4):612‐622. 25817016 10.1016/j.ajhg.2015.02.015 PMC4385180

[5] White JJ , Mazzeu JF , Hoischen A , et al. DVL3 alleles resulting in a ‐1 frameshift of the last exon mediate autosomal‐dominant Robinow Syndrome. Am J Hum Genet. 2016;98(3):553‐561. 26924530 10.1016/j.ajhg.2016.01.005 PMC4800044

[6] GeneReview: ROR2‐Related Robinow Syndrome. https://www.ncbi.nlm.nih.gov/books/NBK1240. In.

[7] Aglan M , Amr K , Ismail S , et al. Clinical and molecular characterization of seven Egyptian families with autosomal recessive robinow syndrome: Identification of four novel ROR2 gene mutations. Am J Med Genet A. 2015;167A(12):3054‐3061. 26284319 10.1002/ajmg.a.37287

[8] Yang K , Shen M , Yan Y , et al. Genetic analysis in fetal skeletal dysplasias by trio whole‐exome sequencing. Biomed Res Int. 2019;2019:1‐8. 10.1155/2019/2492590 PMC6537022 31218223

[9] Wang R , Tan J , Chen T , et al. ATP13A2 facilitates HDAC6 recruitment to lysosome to promote autophagosome‐lysosome fusion. J Cell Biol. 2019;218(1):267‐284. 30538141 10.1083/jcb.201804165 PMC6314552

[10] Bain MD , Winter RM , Burn J . Robinow syndrome without mesomelic 'brachymelia': a report of five cases. J Med Genet. 1986;23:350‐354. 3746837 10.1136/jmg.23.4.350 PMC1049704

[11] Teebi AS . Autosomal recessive Robinow Syndrome. Am J Med Genet. 1990;35(1):64‐68. 2301471 10.1002/ajmg.1320350112

[12] Afzal AR , Rajab A , Fenske CD , et al. Recessive Robinow syndrome, allelic to dominant brachydactyly type B, is caused by mutation of ROR2. Nature. 2000;25:419‐422. 10.1038/78107 10932186

[13] van Bokhoven H , Celli J , Kayserili H , et al. Mutation of the gene encoding the ROR2 tyrosine kinase causes autosomal recessive Robinow syndrome. Nature. 2000;25:423‐426. 10.1038/78113 10932187

[14] Weissenbock M , Latham R , Nishita M , et al. Genetic interactions between Ror2 and Wnt9a, Ror1 and Wnt9a and Ror2 and Ror1: Phenotypic analysis of the limb skeleton and palate in compound mutants. Genes Cells. 2019;24(4):307‐317. 30801848 10.1111/gtc.12676 PMC7340625

[15] Maeda K , Kobayashi Y , Udagawa N , et al. Wnt5a‐Ror2 signaling between osteoblast‐lineage cells and osteoclast precursors enhances osteoclastogenesis. Nat Med. 2012;18(3):405‐412. 22344299 10.1038/nm.2653

[16] Masiakowski P , Carroll RD . A novel family of cell surface receptors with tyrosine kinase‐like domain. J Biol Chem. 1992;267(36):26181‐26190. 1334494

[17] Oldridge M , Fortuna AM , Maringa M , et al. Dominant mutations in ROR2, encoding an orphan receptor tyrosine kinase, cause brachydactyly type. B. nat Genet. 2000;24:275‐278. 10700182 10.1038/73495

[18] Richards S , Aziz N , Bale S , et al. Standards and guidelines for the interpretation of sequence variants: a joint consensus recommendation of the American college of medical genetics and genomics and the association for molecular pathology. Genet med. 2015;17(5):405‐424. 25741868 10.1038/gim.2015.30 PMC4544753

[19] Ali BR , Jeffery S , Patel N , et al. Novel Robinow syndrome causing mutations in the proximal region of the frizzled‐like domain of ROR2 are retained in the endoplasmic reticulum. Hum Genet. 2007;122(3–4):389‐395. 17665217 10.1007/s00439-007-0409-0

[20] Mehawej C , Chouery E , Maalouf D , et al. Identification of a novel causative mutation in the ROR2 gene in a Lebanese family with a mild form of recessive Robinow syndrome. Eur J Med Genet. 2012;55(2):103‐108. 22178368 10.1016/j.ejmg.2011.11.003

[21] Lara E , Calvanese V , Huidobro C , et al. Epigenetic repression of ROR2 has a Wnt‐mediated, pro‐tumourigenic role in colon cancer. Mol Cancer. 2010;9:170. 20591152 10.1186/1476-4598-9-170 PMC2903502

[22] Basu M , Roy SS . Wnt/beta‐catenin pathway is regulated by PITX2 homeodomain protein and thus contributes to the proliferation of human ovarian adenocarcinoma cell, SKOV‐3. J Biol Chem. 2013;288(6):4355‐4367. 23250740 10.1074/jbc.M112.409102 PMC3567686

[23] Sun B , Ye X , Lin L , Shen M , Jiang T . Up‐regulation of ROR2 is associated with unfavorable prognosis and tumor progression in cervical cancer. Int J Clin Exp Pathol. 2015;8(1):856‐861. 25755786 PMC4348873

[24] Debebe Z , Rathmell WK . Ror2 as a therapeutic target in cancer. Pharmacol Ther. 2015;150:143‐148. 25614331 10.1016/j.pharmthera.2015.01.010 PMC9236825

